# Dementia Specialists’ Perspectives on Improving Rural Veterans’ Access to Care

**DOI:** 10.1093/geroni/igaf122.3745

**Published:** 2025-12-31

**Authors:** Sarah McDannold, Brittany Anderson, Tammy Walkner, Michelle Hilgeman, Hilary Mosher, Lauren Moo, Guneet Jasuja, Heather Davila

**Affiliations:** VA Bedford Healthcare System, Bedford, Massachusetts, United States; Iowa City VA Health Care System, Iowa City, Iowa, United States; Iowa City VA Health Care System, Iowa City, Iowa, United States; Tuscaloosa VA Medical Center, Tuscaloosa, Alabama, United States; Minneapolis VA Medical Center, Minneapolis, Minnesota, United States; VA Bedford Healthcare System, Bedford, Massachusetts, United States; Center for Health Optimization and Implementation Research, VA Bedford, Boston, Massachusetts, United States; Iowa City VA Healthcare System, Iowa City, Iowa, United States

## Abstract

Outside the Veterans Health Administration (VHA), rurality-based differences in dementia care access have been reported (e.g., fewer physician and home health visits among rural vs. urban residents). However, this has not been examined within the VHA, which prioritizes rural healthcare access. To address this gap, we conducted semi-structured interviews with dementia specialists (N = 20) at five rural VHA medical centers in two regions. Based on Drebig’s “pathways to dementia care” framework, interviews focused on understanding gaps and strategies to meet rural Veterans’ care needs at three stages: dementia warning signs; evaluation and diagnosis; and aligning treatment and services to optimize patient and caregiver quality of life. We analyzed the thematic content of interviews using rapid qualitative analysis. Common gaps reported were 1) need for earlier recognition of dementia warning signs to access services; 2) lack of dementia specialists, particularly in the community; 3) limited resources to support family caregivers; and 4) limited availability of supportive services such as home care, respite, and residential long-term care. Common strategies to address gaps included 1) education and outreach to primary care clinicians; 2) tele-consult services; and 3) care transitions programs for Veterans with challenging behavioral symptoms. Clinicians consistently described VHA as having more robust dementia specialty care services than what was available in the community. Considerable gaps remain in rural access to dementia care around recognition and evaluation of dementia, and coordination and availability of supportive services. Optimizing access to dementia-related services for rural Veterans requires tailored approaches to leverage VHA and community resources.

